# Can Unmet Needs Be Addressed by Adjunctive Therapies? Findings from a Patient Perspectives Survey in Adults with Type 1 Diabetes

**DOI:** 10.1177/23743735241257811

**Published:** 2024-05-25

**Authors:** Bella D. Lamaro, Jerry R. Greenfield, Jennifer R. Snaith

**Affiliations:** 1 Faculty of Medicine and Health, University of New South Wales, Sydney, New South Wales, Australia; 2 Department of Diabetes and Endocrinology, St Vincent's Hospital, Sydney, New South Wales, Australia; 3 Diabetes and Metabolism, 2785 Garvan Institute of Medical Research, Sydney, New South Wales, Australia

**Keywords:** type 1 diabetes, glucagon-like peptide-1 receptor agonist, GLP-1, adjunctive therapy, patient preferences, survey, non-insulin medications

## Abstract

Many individuals with type 1 diabetes (T1D) do not achieve their management goals. The patient perspective on unmet needs in T1D may guide the role of adjunctive therapies, including glucagon like peptide-1 receptor agonists (GLP-1RAs). A quantitative online survey (n = 133) assessed (1) self-reported demographic and management data, (2) management priorities, satisfaction, and willingness to use adjunctive therapies and (3) conducted a risk-benefit analysis using three masked drug profiles (1.8 mg vs 0.6 mg liraglutide vs placebo). A subgroup of respondents (n = 20) participated in semi-structured interviews to extend upon survey insights. Needs were unmet by current treatment in 28% of surveyed individuals. The greatest unmet needs included (1) glycemia, (2) management-related fatigue, and (3) weight management. Most respondents (94%) indicated that they would use adjunctive therapies. The preferred administration route was daily tablets (66%) followed by weekly injections (32%). Metabolic improvements were most valued (reduction in hypoglycemia, hyperglycemia). Most respondents (94%) preferred the liraglutide risk-benefit profile (1.8 mg, then 0.6 mg) over placebo. Individuals with T1D self-report many unmet needs. While not currently approved in T1D, GLP-1RA properties align with many management priorities reported by individuals with T1D.

## Introduction

The management of type 1 diabetes (T1D) necessitates lifelong insulin-replacement. Despite therapeutic advances, many patients experience suboptimal glycemia and vascular complications.^1,2^ Individuals with T1D have a greater risk of cardiovascular disease (CVD) than the general population.^3,4^ Intensive insulin therapy increases risk of hypoglycemia, and induces insulin resistance and weight gain, partially counteracting the benefits of strict glucose control.^4‐6^ Insulin-induced weight gain and excess body weight exacerbates insulin resistance and increases insulin requirements, metabolic syndrome features and microvascular and macrovascular diabetes-related complications.^4,5,7,8^ Metabolic syndrome, characterised by central obesity, hypertension, insulin resistance and atherogenic dyslipidemia, has been estimated to affect 23.7% of people living with T1D.^7^ Adjunctive therapies in T1D may address these management issues. Understanding the priorities and unmet needs of individuals with T1D is essential to optimize diabetes management, improve patient outcomes and guide design of research trials in T1D.

Currently approved for type 2 diabetes (T2D) and obesity, glucagon-like peptide-1 receptor agonists (GLP-1RAs) are promising in T1D treatment considering their insulin-independent glucose-lowering properties and cardiometabolic benefits.^4^ While GLP-1RAs are not approved for use in T1D, there is an emerging evidence base, particularly for overweight and obese individuals with T1D with inadequate glycemic control.^6^ A real-world, global study found 13% of individuals with T1D have taken a non-insulin adjunct for glycemic management, and just 1.7% were prescribed a GLP-1RA off-label.^9^ Potential benefits for individuals with T1D include decreased HbA1c, improved glycemic stability, weight reduction, and improved insulin sensitivity and cardiovascular health.^10,11^

Liraglutide is a GLP-1RA that is given as a daily subcutaneous injection and has been assessed in early-stage clinical trials in T1D.^8,12‐16^ Compared to placebo, liraglutide used adjunctively with insulin resulted in dose-dependent reduction in bodyweight^8,12‐18^ and adiposity^15^ while conserving lean mass,^14^ increased time in normal glucose range,^14,17^ and reduction in HbA1c,^12‐14,17,19^ total daily insulin (TDI),^8,12,13,16‐18^ and glycemic variability.^18^

It is unclear whether the cardiometabolic benefits of GLP-1RAs observed in T2D are also experienced in T1D. Some liraglutide studies in T1D reported decreased systolic and diastolic blood pressure (BP),^14‐16^ suggesting potential cardiovascular effects. While dose-dependent gastrointestinal side-effects are common across different GLP-1RA formulations,^8,12,13,16,18,20‐22^ multiple T1D trials reported no serious drug-related adverse events with GLP-1RAs, including pancreatitis, hepatic damage, or hypoglycemic episodes requiring medical intervention.^13,21,22^

Although clinical trials have examined metabolic effects of GLP-1RAs in T1D, it is important to consider patient acceptability on GLP-1RA use. While studies have assessed patient perspectives on sodium–glucose cotransporter 2 (SGLT-2) therapies used adjunctively with insulin in T1D,^23,24^ no study has assessed patient perspectives on adjunctive GLP-1RAs.

Evidently, there is a need to assess whether adjunctive therapies are valued by individuals with T1D. This study had two main objectives: (1) to investigate patient needs, preferences, and priorities for T1D management, and (2) to assess, from the patient perspective, the utility, suitability, and viability of GLP-1RAs as an adjunct to insulin therapy in T1D.

## Methods

### Study Design and Participants

This study had two components (Supplement 1). Study A was a quantitative online survey to identify treatment priorities, unmet needs, and preferences in T1D. Study B consisted of semi-structured individual interviews of a subgroup of survey participants to further explore management priorities, and whether GLP-1RAs may align with these priorities.

### Study A: Quantitative Online Survey

Adults with T1D (≥18 years old with a self-reported T1D diagnosis) were invited to complete an anonymous quantitative online survey via Facebook diabetes forums and clinic databases from St Vincent's Hospital Sydney and Endocrinology private consulting rooms. Participants provided written informed consent. The survey was available for eight weeks. Study data were managed using REDCap (Research Electronic Data Capture).^25,26^ The full survey is provided in Supplement 2.

The survey (1) captured self-reported demographic, metabolic and diabetes management data, (2) assessed current disease burden, management priorities, satisfaction, and willingness to use adjunctive therapy using Likert scales, (3) generated an unmet-needs score by subtracting importance scores from satisfaction scores, and (4) conducted a risk-benefit conjoint analysis by presenting participants with three adjunct profiles masked to drug, route and dose (profiles provided in Supplement 3). Efficacy and side-effect data for these adjunct profiles were derived from phase-three trials of 1.8 mg and 0.6 mg liraglutide and placebo in T1D.^12,13^

Upon survey completion, respondents were invited to participate in individual interviews for Study B.

### Study B: Semi-Structured Interviews

Semi-structured individual interviews were conducted and transcribed via video-conferencing (Microsoft Teams, Version 1.6.00.24078). Interview questions are provided in Supplement 4. The relative importance of risks and benefits was quantitatively assessed using a point-allocation exercise involving a fixed ‘budget’ of 100 points.

Transcripts were coded by themes. Recruitment continued until thematic saturation was achieved and no new insights emerged.

### Statistical Procedures

IBM SPSS Statistics (Version 27.0) was used for survey subgroup analyses. Binary subgroups of interest included: body mass index (BMI) (<25 kg/m^2^ vs ≥25 kg/m^2^), HbA1c (≤7.0% vs >7.0%), insulin delivery system (multiple daily injections (MDI) versus continuous subcutaneous insulin infusion (CSII)), use of non-insulin adjunct (yes vs no), and presence of comorbidities (heart disease, hypertension, and/or hyperlipidemia; yes vs no). Likert scale values were translated into numerical, ranked values (0 to 4) and treated as ordinal data.

Normality was determined using Kolmogorov-Smirnov and Shapiro-Wilk tests paired with visual inspection of histograms. Between group differences were assessed with an independent unpaired t-test (normally distributed and/or continuous data), or a Mann-Whitney U test (non-normally distributed and/or ordinal data). For comparison of binary variables, a chi-squared test was used. P-value significance was set at p < 0.05.

Normally distributed continuous data was represented as mean ± standard deviation (SD), non-normally distributed continuous data as median [25^th^ centile, 75^th^ centile], and ordinal data was represented with p-values only. Missing data was not imputed.

## Results

### Study A: Survey

#### Participant Characteristics

There were 133 survey respondents (Table 1, Supplement 1). The cohort had long duration diabetes. The majority (60.5%) used multiple daily injections (MDI) as their insulin delivery method, and almost all (90.7%) used continuous glucose monitoring (CGM). The mean BMI was 26.4 ± 5.7 kg/m^2^, with 30.5% of respondents classified as overweight and 21.9% as obese.

**Table 1.** Participant Demographic Data.

Parameter		Respondents (n = 133)
**Age **(years)		51.0 ± 14.5
**Diabetes duration **(years)		25.7 ± 16.7
**Gender **(n, %)	Female	73 (56.6)
	Male	56 (43.4)
**Location Type** (n, %)	Metropolitan	104 (83.9)
	Rural	19 (15.3)
	Remote	1 (0.8)
**BMI **(kg/m^2^)	Mean BMI	26.4 ± 5.7
	<25.0: n (%)	61 (47.7)
	25.0 to 29.9: n (%)	39 (30.5)
	>30.0: n (%)	28 (21.9)
**Mean HbA1c** (%, mmol/mol)		7.1 (54) ± 1.2 (13)
**Insulin Delivery Method** (n, %)	Multiple daily injections (MDI)	78 (60.5)
	Continuous subcutaneous insulin infusion (CSII)	51 (39.5)
**BGL Monitoring** (n, %)	Continuous glucose monitoring (CGM)	117 (90.7)
**Diabetes-related complications** (n, %)	Retinopathy	28 (22)
	Lower limb neuropathy	14 (11)
	Nephropathy	4 (3)
	Heart disease	14 (11)
	Hypercholesterolemia	39 (30)
	Hypertension	30 (23)
	Depression and/or anxiety	35 (27)

Continuous data presented as sample mean ± standard deviation. Categorical data presented as numerical count (percentage). Gender identity self-reported by participants. See Supplementary 1 for details regarding survey completion numbers for Screening Survey and Section 1.

Abbreviations: BGL, blood glucose level; BMI, body mass index; HbA1c, glycated hemoglobin; n, sample size.

#### Management Priorities, Satisfaction and Unmet Needs

**Figure 1.**
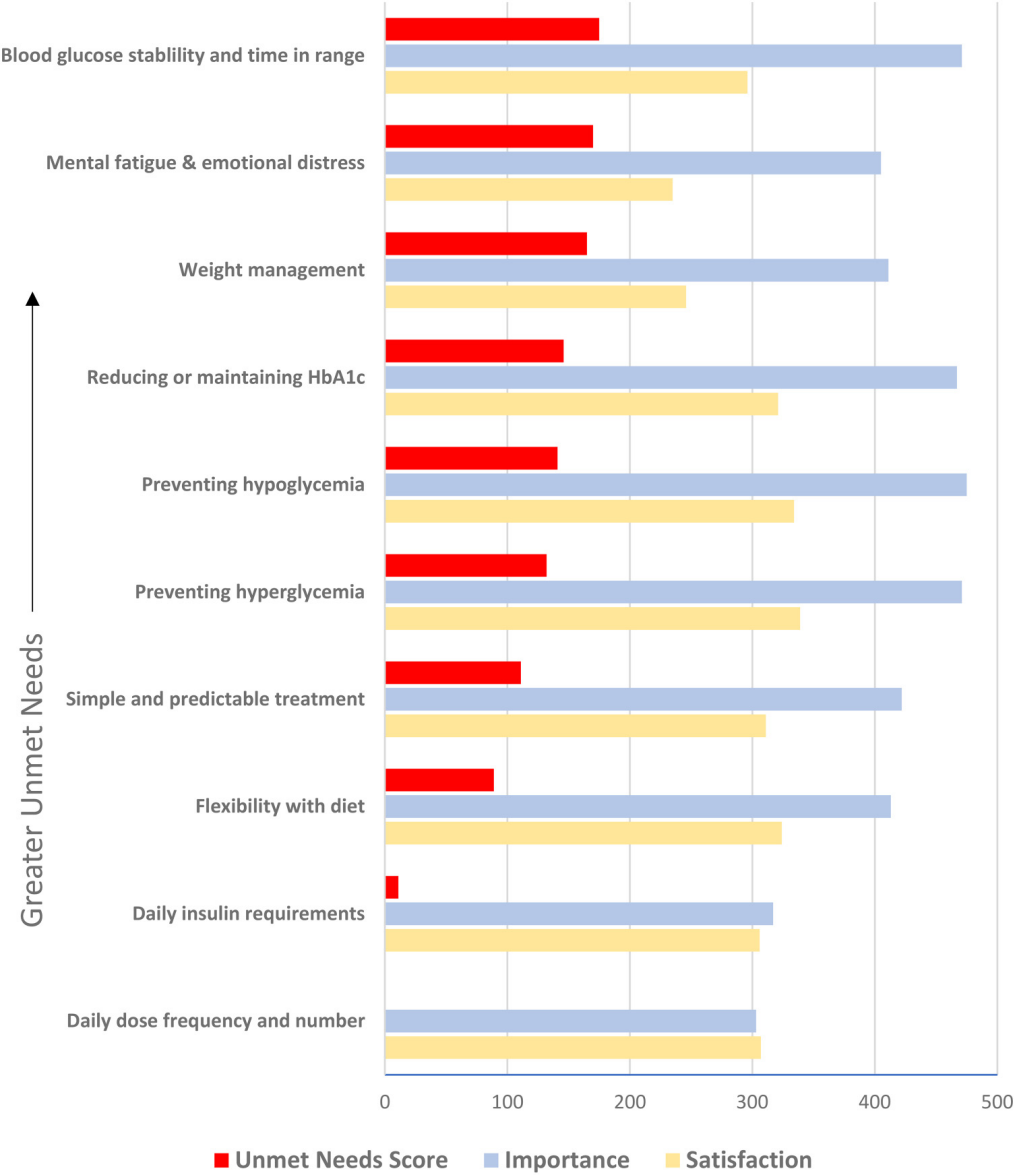
**Priorities, Satisfaction and Unmet Needs in Type 1 Diabetes Management. **Survey responses for management priorities and satisfaction were translated into numerical results on a Likert scale (0 = not at all important/satisfied, 4 = completely important/satisfied). The most important management goals were (1) preventing hypoglycemia, (2) preventing hyperglycemia/keeping blood glucose levels stable/increasing time-in-range, and (3) reducing/maintaining HbA1c. Respondents were least satisfied with (1) levels of mental fatigue/emotional distress, (2) prevention of weight gain and (3) blood glucose levels stability and time in ideal range. The unmet needs score was calculated as the difference between importance and satisfaction scores, representing priority areas for improvement. Management attributes are arranged by unmet needs scores in descending order. The greatest unmet needs were (1) improving blood glucose level stability and time-in-range, (2) reducing management-related mental fatigue and emotional distress and (3) prevention of weight gain. Abbreviations: HbA1c, glycated hemoglobin.

The most important management goals related to glycemia, including (1) preventing hypoglycemia, (2) preventing hyperglycemia and (3) increasing time in ideal glucose range (Figure 1). Respondents were least satisfied with mental fatigue while managing diabetes, followed by weight management, blood glucose stability and time spent in target glucose range. The greatest unmet needs scores were (1) improving blood glucose stability and time-in-range (TIR), (2) reducing management-related mental fatigue and (3) prevention of weight gain. Twenty-eight percent indicated that their needs were not met by their current treatment.

#### Willingness to Use an Adjunct

Most respondents (94%) indicated they would consider adjunctive therapy to better manage their diabetes. Of these individuals, 99% accepted a tablet whereas 84% accepted an injection. The preferred administration method was a daily tablet (65.9%), followed by weekly injections (32%). Of those who preferred a daily tablet, 84% selected a weekly injection as their second preference. Of those who preferred a weekly injection, 79% preferred a daily tablet as their second choice, and 18% preferred a daily injection.

#### Adjunct Attribute Preferences

**Figure 2.**
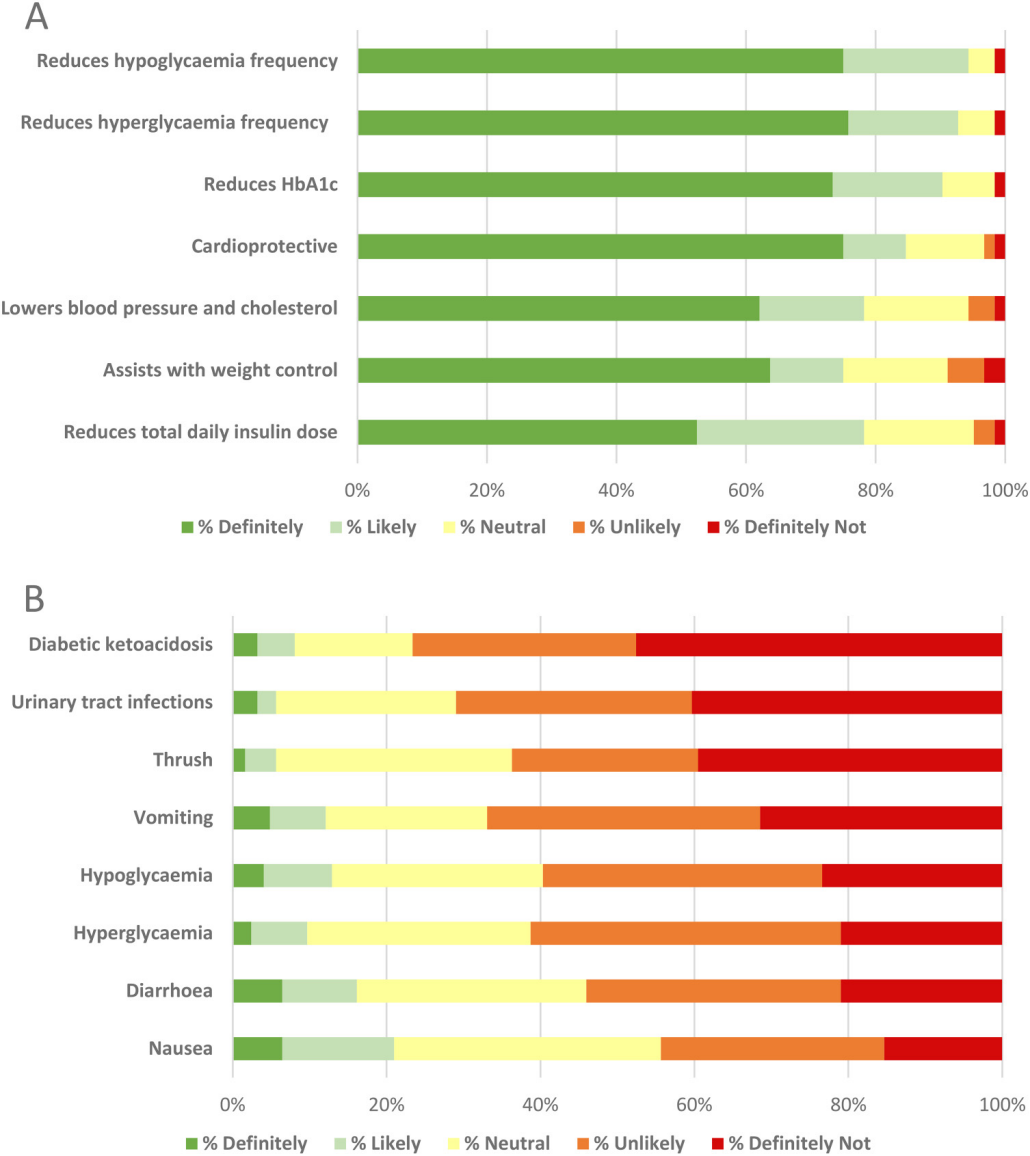
**Likelihood of using adjunct according to potential benefits and adverse effects**. Adjunct benefits and risks were derived from known benefits of GLP-1RAs in T1D, in addition to known risks of other adjuncts (SGLT-2 inhibitors, Metformin). (A) Regarding benefits, respondents were inclined towards improving glycemia, weight management and cardiovascular health. Benefits are ordered according to sum of ‘definitely’ scores weighted ×1.5 and ‘likely’ weighted ×1. (B) Regarding potential adverse-effects, diabetic ketoacidosis and infective adverse effects were most deterring, followed by concerns around hyper- and hypoglycemia, and gastrointestinal symptoms. Adverse effects are ordered according to sum of ‘definitely not’ scores weighted ×1.5 and ‘unlikely’ weighted ×1. Abbreviations: GLP-1RA, glucagon-like peptide-1 receptor agonist; HbA1c, glycated hemoglobin; SGLT-2, sodium–glucose cotransporter 2; T1D, type 1 diabetes mellitus.

Glycemic improvements were most valued by participants, with 95%, 93% and 90% indicating they would ‘likely’ or ‘definitely’ use an adjunct if it reduced hypoglycemia frequency, hyperglycemia frequency, and HbA1c, respectively (Figure 2A). Regarding cardioprotective properties, 85% and 78% of participants indicated they would ‘likely’ or ‘definitely’ use an adjunct that offers protection from heart disease and lower BP and/or cholesterol, respectively. Weight management was valued by 75% of respondents indicating their preference to use an adjunct that assists with weight control. Diabetic ketoacidosis (DKA), urinary tract infections and vomiting were most deterring to participants, with 77%, 71% and 67% indicating aversion to using an adjunct with these adverse-effects, respectively (Figure 2B).

#### Conjoint Risk-Benefit Analysis

**Figure 3.**
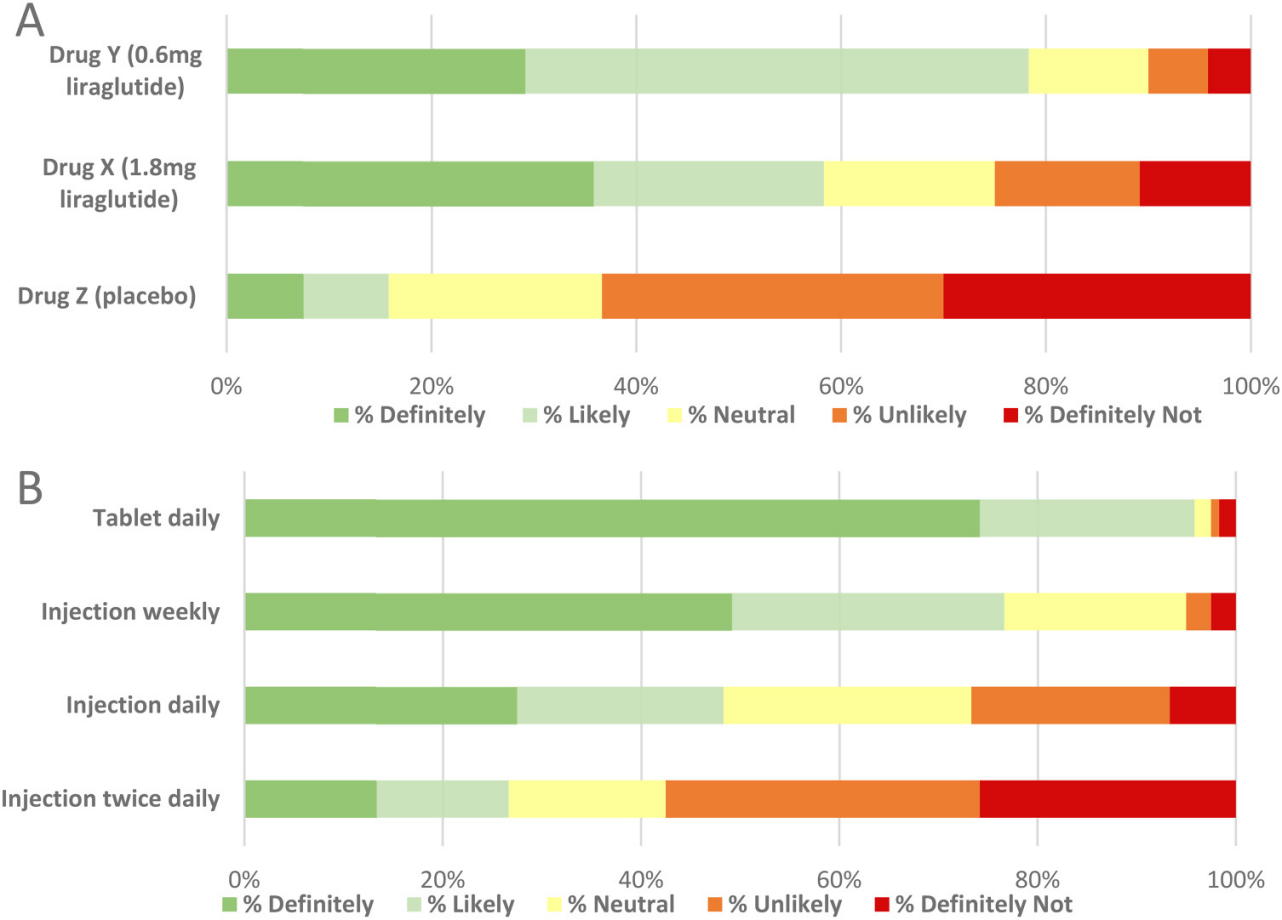
**Conjoint benefit-risk analysis using three masked drug profiles. **Efficacy and side-effect data for the masked profiles were derived from phase-three trials of 1.8 mg liraglutide (Drug X), 0.6 mg liraglutide (Drug Y) and placebo (Drug Z) in T1D.^12, 13^ Administration options mirror currently available GLP-1RA administration routes. (A) Participants were most likely to use 0.6 mg liraglutide. There was a mixed response to 1.8 mg liraglutide. Nearly two-thirds (63%) indicated they would ‘likely’ or ‘definitely’ not use the placebo profile. (B) The likelihood of using their preferred adjunct profile was greatest as a daily tablet, followed by weekly injection. There was minimal aversion to these routes. The twice-daily injection was least favored with the majority (57.5%) indicating reluctance to use their preferred adjunct via this route. Abbreviations: T1D, type 1 diabetes mellitus; GLP-1RA, glucagon-like peptide-1 receptor agonist; HbA1c, glycated hemoglobin; mg, milligram.

When asked to choose between three masked drug profiles (Supplement 3), most respondents (94%) preferred the risk-benefit profile of liraglutide over placebo, with 48% and 47% selecting 1.8 mg and 0.6 mg liraglutide, respectively, as their first preference. Most participants indicated they would ‘very likely’ or ‘definitely’ use the masked 0.6 mg and 1.8 mg (78% and 58%, respectively) liraglutide as an adjunct to insulin (Figure 3A).

When asked about their likelihood of using their preferred masked drug according to administration route, 96% and 77% of participants indicated they would ‘likely’ or ‘definitely’ use a tablet and weekly injection, respectively. Conversely, 58% and 27% of participants indicated they would ‘likely not’ or ‘definitely not’ use their preferred adjunct if it was a twice-daily and once-daily injection, respectively (Figure 3b).

### Subgroup Analysis

#### Body Mass Index (BMI): BMI ≥25 kg/m^2^ Vs BMI <25 kg/m^2^

The median HbA1c was significantly greater in the BMI ≥25 kg/m^2^ (n = 67) than BMI <25 kg/m^2^ (n = 61) group (7.4[6.8, 8.0]% vs 6.5[6.0, 7.3]%, p < 0.0001). Individuals with BMI ≥25 kg/m^2^ were less likely to achieve an HbA1c ≤7.0% (38% vs 73% with HbA1c ≤7.0%, BMI ≥25 kg/m^2^ vs <25 kg/m^2^ respectively; p < 0.0001) and were less satisfied with their HbA1c (p = 0.018).

Individuals with BMI ≥25 kg/m^2^ were less satisfied with their current management overall than individuals with a BMI <25 kg/m^2^ (36% vs 18% unsatisfied, BMI ≥25 kg/m^2^ vs <25 kg/m^2^ respectively; p = 0.028), and less satisfied with weight management (p < 0.0001).

Those with BMI ≥25 kg/m^2^ displayed a greater inclination to use an adjunct that assists with weight (p < 0.0001), BP and cholesterol reduction (p = 0.002), and cardiovascular protection (p = 0.017).

#### Glycated Hemoglobin (HbA1c): HbA1c ≤7.0% Vs HbA1c >7.0%

Compared to individuals with HbA1c ≤7.0% (n = 69), individuals with HbA1c>7.0% (n = 58) reported a higher frequency of weight gain/management issues (p = 0.041) and lower HbA1c satisfaction (p < 0.0001), while placing less importance on increasing TIR (p = 0.047) and optimizing HbA1c (p = 0.002).

#### Non-insulin Adjuncts: Individuals Currently Prescribed Adjunct vs Insulin Monotherapy

For individuals currently taking a non-insulin adjunct (n = 20), BMI was significantly higher compared to those taking insulin only to manage their diabetes (n = 107) (29.4 [25.4, 31.6] kg/m^2^ vs 24.7 [22.3, 27.2] kg/m^2^, p = 0.004). Individuals using an adjunct reported higher frequency of weight management issues (p = 0.002), mental fatigue (p = 0.024) and finding management overly time consuming (p = 0.026). Individuals using an adjunct reported lower satisfaction for weight management on current treatment (p < 0.001), but higher importance for weight management (p = 0.007), diet flexibility (p = 0.035), and improving mental fatigue/emotional distress (p = 0.032) than those taking insulin only.

Individuals currently using a non-insulin adjunct showed a greater inclination to use an adjunct that reduces TDI (p = 0.047) or HbA1c (p = 0.031) or assists with weight control (p = 0.003). Simultaneously, these individuals showed a higher risk tolerance, reflected in a greater likelihood of using adjuncts with risks including nausea (p = 0.002), hypoglycemia (p = 0.019) and DKA (p = 0.011).

#### Comorbid status: Heart Disease, Hypertension and Hyperlipidemia

Individuals with heart disease (n = 14) were more likely to desire an adjunct that assists with weight control (p = 0.004) or with cardioprotection (p = 0.024) than those without heart disease (n = 110).

Individuals with hypertension (n = 30) had a higher ranked likelihood of using a cardioprotective adjunct (p = 0.024) or a BP and cholesterol lowering adjunct (p = 0.001) than those without hypertension (n = 94).

Individuals with hyperlipidemia (n = 38) were more likely to desire an adjunct that reduces BP and cholesterol (p = 0.026) than those without hyperlipidemia (n = 86).

### Study B: Interviews

#### Quantitative

Overall, increasing glucose stability and TIR was most prioritized (Supplement 5) with 15/20 participants allocating this attribute the highest point score (data not shown). From aggregated attribute scores, benefits ranked higher than all risks.

#### Qualitative

While each participant had unique experiences and preferences regarding diabetes management, major themes emerged:

##### Priorities in Diabetes Management

While participants all prioritized keeping blood glucose levels stable, many emphasized the importance of non-glycemic goals including managing weight, overall health, and emotional wellbeing:

*“I want to maintain a stable blood sugar level as much as possible.” (Participant 1, age 43, diagnosed at age 25, high blood pressure and cholesterol)*

*“My main goal is to avoid complications.” (Participant 2, age 47, diagnosed at age 8)*

*“I focus on living a normal life while keeping my diabetes in check.” (Participant 3, age 25, diagnosed at age 6, depression)*

*“I try and manage my weight as much as I can, that's probably the biggest problem that I've got.” (Participant 6, age 64, diagnosed at age 13, heart disease, high blood pressure, diabetic retinopathy)*

##### Satisfaction with Current Treatment

Participants reported varying degrees of satisfaction with their diabetes management. Some expressed contentment:

*“I'm quite satisfied with my current treatment plan.” (Participant 1, age 43, diagnosed at age 25, high blood pressure and cholesterol)*

*“It's a bit of a struggle, but overall, I'm okay with my treatment.” (Participant 3, age 25, diagnosed at age 6, depression)*

Many expressed frustration at the lack of simplicity and predictability of treatment:

*“I just can't manage to keep myself stable. I go up and down, up and down. It's always up and down.” (Participant 6, age 64, diagnosed at age 13, heart disease, high blood pressure, diabetic retinopathy)*

*“Some days I'm perfect, other days I'm up, down, round and round.” (Participant 7, age 57, diagnosed at age 13, high blood pressure)*

*“You tried the hardest you can… you're doing the best you can and sometimes when you go into appointments and you're still not good enough, what about this, why aren’t you doing this, why? And it's just like, I am trying so hard.” (Participant 9, age 33, diagnosed at age 13)*

##### Willingness to Explore Adjunct Medications

Participants were generally open to trying additional medications in addition to insulin if they offered clear benefits:

*“If it offers me benefits that are appreciable enough.” (Participant 12, age 72, diagnosed at age 68)*

*“It depends on the benefit. If it's something that overall simplifies the management and takes away the concerning aspects of it and the stress, then yes, that it will be for sure a yes.” (Participant 19, age 41, diagnosed at age 37)*

There was interest in medications that improve glycemia, reduce weight, and have cardiovascular benefits. Preference for TDI reduction varied, with greater importance placed on reducing TDI by individuals requiring larger quantities of insulin (over 1 unit of insulin per kilogram of bodyweight) than for individuals on lower daily doses:

*“The main factor [for using an adjunct] would be the outcome in terms of those metrics that we look at in terms of diabetes management outcomes, so time-in-range and HbA1c, which in theory, should drive the probability of having complications long term.” (Participant 19, age 41, diagnosed at age 37)*

*“After talking with [my endocrinologist] in my last appointment, we were starting to believe that I have some sort of insulin resistance… I'd want to see that HbA1c go down, I’d want to see insulin dosages down, I'm on too much insulin now. And yeah, sure, if I can lose weight, and that's going to help with the insulin dose as well.” (Participant 15, age 32, diagnosed at age 5, high blood pressure and cholesterol, diabetic retinopathy)*

*“To be honest, [reducing TDI] is neither here nor there for me, like that's not an important factor for me.” (Participant 9, age 33, diagnosed at age 13)*

*“I really don’t mind [about reducing TDI]. I’m not on a big dose.” (Participant 10, age 79, diagnosed at age 27, high blood pressure and cholesterol)*

##### Risk and Side-Effect Tolerance

Participants were willing to tolerate mild and transient side-effects if the benefits outweighed the discomfort. However, prolonged impact on quality of life would not be tolerated:

*“I just look at like, is this side-effect going to be a short-term thing? And if it's not, is it something I can manage well on a long-term basis.” (Participant 3, age 25, diagnosed at age 6, depression)*

*“I don't think it would be specific side-effects, it would be the severity of the side-effects that would be a problem. If they were relatively low grade, I could live with that, if they were interfering with my quality of life or stopping me doing things, then I probably wouldn't [take the adjunct].” (Participant 18, age 56, diagnosed at age 55, high cholesterol)*

Interviewees generally accepted an increased risk of hyperglycemia, hypoglycemia, and ketoacidosis due to perceived ability to manage these events, particularly if benefits outweigh risks:

*“I don't see them [hyperglycemia, hypoglycemia, DKA] as risks that I would be uncomfortable with because then I know that I can adjust.” (Participant 17, age 47, diagnosed at age 12, heart disease, high blood pressure and cholesterol, anxiety)*

*“[Hypoglycemia or DKA] would be something to monitor for, it wouldn't put me off.” (Participant 9, age 33, diagnosed at age 13)*

Participants who had previous DKA were highly deterred by the risk of another event, unlike individuals who had never experienced DKA:

*“See risk of diabetic ketoacidosis… that's not important to me cause I've never gotten that.” (Participant 5, age 59, diagnosed at age 3, high blood pressure and cholesterol, depression, diabetic retinopathy)*

*“I’ve had [DKA] once in my life. That would be massively deterring. Avoiding DKA really is more important than reducing the insulin.” (Participant 7, age 57, diagnosed at age 13, high blood pressure)*

*“Right before I was diagnosed, I went into diabetic ketoacidosis… I don't ever want to do that again.” (Participant 16, age 56, diagnosed at age 20, diabetic retinopathy, diabetic neuropathy, high blood pressure)*

Notably, participants satisfied with current management had lower tolerance for side-effects:

*“I think [people struggling with diabetes management] would probably jump at [an adjunct], whereas I think people like me who are living fairly unchallenged and comfortable and have got good control, I imagine, would only jump at them if they came without too many of their own [side-effects].” (Participant 12, age 72, diagnosed at age 68)*

##### Preferences for Administrative Route

While most participants were open to both a weekly injection and daily tablet, most chose oral tablets as their preferred administrative route due to its simplicity, and non-invasive nature:

*“Injections are a hassle, so a tablet would be my choice.” (Participant 2, age 47, diagnosed at age 8)*

*“A tablet, I’ve had enough injections in my time.” (Participant 11, age 67, diagnosed at age 42, diabetic retinopathy)*

*“Probably daily tablet for me, because there’d probably be a good chance I'd forget the weekly injection.” (Participant 7, age 57, diagnosed at age 13, high blood pressure)*

The likelihood of adherence tablets was considered improved by those with an existing routine, but was a concern to participants with a large tablet burden:

*“I already take other medications that are once a day, so that's quite easy for me to do.” (Participant 3, age 25, diagnosed at age 6, depression)*

*“I'd choose a tablet, just put it in with the ones I already take.” (Participant 5, age 59, diagnosed at age 3, high blood pressure and cholesterol, depression, diabetic retinopathy)*

*“I'm sick and tired of tablets because I'm taking so many every day, so I’d prefer a weekly injection, because I'm taking so many tablets [over 15 daily] that I don’t want to take another one.” (Participant 10, age 79, diagnosed at age 27, high blood pressure and cholesterol)*

The weekly injection was also favorable due to convenience and familiarity:

*“A weekly injection seems very doable. You just do it once and forget about it. And the fact that it's an injection wouldn't typically scare a diabetic because they're very used to that anyway.” (Participant 1, age 43, diagnosed at age 25, high blood pressure and cholesterol)*

*“I probably would go with the injection. I mean, I certainly have a lot of experience with giving them.” (Participant 8, age 63, diagnosed at age 17, high blood pressure)*

##### Impact of Non-insulin Adjuncts

A few participants were already taking non-insulin adjuncts for diabetes, including metformin, empagliflozin and semaglutide. These individuals noticed significantly improved BGL stability and reduced TDI, HbA1c, and weight:

*“[Empagliflozin/metformin] has dramatically reduced my total daily intake of insulin from 100 down to 60 units per day.” (Participant 1, age 43, diagnosed at age 25, high blood pressure and cholesterol)*

*“I think [metformin] is great. Like, I forgot one morning to take it and it was just amazing the difference that it made. Like the sugars were just so, like high. And they just would not come down. So yeah, definitely recommend it.” (Participant 9, age 33, diagnosed at age 13)*

*“[Semaglutide and empagliflozin/metformin] reduced my insulin dose because I was taking over 100 units of insulin a year ago. So that's come down by 20 units, and my blood sugar control’s gotten better [from HbA1c above 9 to now 6.3]. The weight has gradually come off over the year, 15kgs in total … I was very unsatisfied before I started these treatments, and now I'm very satisfied with what's going on.” (Participant 16, age 56, diagnosed at age 20, diabetic retinopathy, diabetic neuropathy, high blood pressure)*

Participants expressed frustration with the challenges of accessing non-insulin adjuncts and significant cost, believing that adjuncts should be subsidized and made available to individuals with T1D, especially if they exhibit insulin resistance:

*“With the results that I'm getting, I'm a bit frustrated about all the hoops you have to jump through and the amount of money you have to pay. Clearly I've got type 1 and a form of type 2 in terms of insulin resistance. So yeah, that frustrates me that it’s only indicated right now for obesity and type 2 diabetes and not type 1.” (Participant 1, age 43, diagnosed at age 25, high blood pressure and cholesterol)*

*“I think that [adjuncts not subsidized for T1D] is a travesty and wrong because if it helps the type 1 diabetic because they have symptoms of type 2, insulin resistance, then clearly they should be made available to the type 1 diabetic. And I'm anecdotally proof because it's improved my blood sugar results quite drastically both of them, from being consistently in the highs, if not over 9 on HbA1c to now being below 7.” (Participant 16, age 56, diagnosed at age 20, diabetic retinopathy, diabetic neuropathy, high blood pressure)*

## Discussion

This study explored the priorities, satisfaction and unmet needs of people living with T1D, and their perceptions regarding the suitability of adjunctive therapies. Survey participants were both willing and likely to use an adjunct to assist with their diabetes management, particularly for potential glycemic, metabolic, and cardiovascular benefits.

The prioritization of glycemic goals mirrored findings in previous studies.^23,24^ While glycemic goals were ranked as important, we found poor satisfaction and a desire for adjunctive therapy that improves glycemia. The low satisfaction and high unmet-needs scores of both glycemic stability/TIR and mental fatigue/emotional distress may be interrelated; numerous studies highlight an association between suboptimal glycemia, increased emotional distress and low diabetes-related satisfaction, while individuals who achieve HbA1c targets demonstrate higher treatment satisfaction and lower hypoglycemia fear.^27‐31^

Participants valued adjuncts that assist with cardioprotection, blood pressure, cholesterol and weight management. Respondents appeared aware of the increased cardiovascular risks associated with T1D. In this study, three-quarters of participants were inclined to use an adjunct for weight management.

Potential side-effects significantly influenced the acceptability and uptake of adjunctive therapies, particularly gastrointestinal, glycemic, and urinary tract infection risks. While these deterred many, risks appeared acceptable if outweighed by benefits. Gastrointestinal side-effects with GLP-1RAs deterred many participants, particularly if ongoing or severe. Counselling that gastrointestinal side-effects may be transient and most pronounced on dose initiation and titration only may overcome potential adherence issues.

Like other studies,^23,24^ risk of diabetic ketoacidosis, urinary sepsis and thrush was highly deterring, whereas hyperglycemia and hypoglycemia were not. Therefore for some, GLP-1RAs may be more suitable than SGLT-2is,^32^ which are associated with higher rates of these adverse effects. Interviews revealed that prior experience of ketoacidosis was a contributing factor to ketoacidosis risk aversion.

Less invasive administration methods and minimizing dose frequency were valued. Once-weekly and oral GLP-1RA formulations offer dosage route and frequency options, potentially increasing treatment compliance and satisfaction compared to daily injected preparations.^33^ The popularity of the daily tablet may reflect its convenience and pain-free administration. The PIONEER clinical trials demonstrated that daily oral semaglutide was comparable to weekly subcutaneous semaglutide,^34,35^ and may offer an option for patients preferring a tablet. Conversely, twice-daily injections were unpopular, indicating dose frequency may affect uptake, though this response may have been influenced by the availability of more favorable alternatives within our survey design.

Adherence and treatment satisfaction may be influenced by an individual's medication routine. As with other studies,^23^ current daily tablet use was associated with increased willingness to take an oral medication. This highlights the impact of an individual's current medication burden on decision-making.

The association between higher BMI and higher HbA1c may be explained by insulin resistance secondary to excess adiposity, and subsequent need for greater insulin doses to maintain ideal glycemia.^4‐6^ Higher insulin doses increase treatment complexity, and may impact glycemic stability. For individuals with BMI ≥25 kg/m^2^, the high dissatisfaction with current management, HbA1c and weight was reflected in the drive to use adjuncts to assist with metabolic goals. Identified as a population with evident unmet needs in both this study and others,^23^ individuals with so-called ‘double diabetes’ may benefit from interventions that assist with weight loss, in order to improve insulin sensitivity, quality of life, and metabolic profile. Overall, individual demographic and clinical factors must be considered to determine appropriateness of adjunct use in T1D.

The perspectives of those currently using adjunct therapies produced valuable insights. This group had a higher BMI than those not using an adjunct, and greatly valued weight management. Interviewed individuals on a non-insulin adjunct reported marked increases in satisfaction in weight, TDI, glycemic stability, HbA1c and quality of life compared to when using insulin only. Studies that review individuals’ perceptions prior to and after using a non-insulin adjunct are warranted.

Our study had some limitations. First, the hypothetical scenarios presented may differ from real-world treatment decisions. Second, the participant sample may not be representative of the general T1D population. The sample was concentrated in metropolitan areas of Australia (New South Wales) and may have skewed towards those managed in the private practice setting. Despite these limitations, the large number of responses, consistency in priorities, concerns and preferences, and alignment with existing literature reinforces our findings.

In summary, individuals with T1D experience many management challenges. Clinical trials suggest that adjunctive GLP-1RAs may assist in achieving important goals in the management of T1D, including optimizing glycemia, reducing weight, increasing insulin sensitivity, and may confer cardio-, nephro- and vascular protection. The findings of this study suggest that individuals with T1D may value GLP-1RAs to assist with their management priorities, goals, and unmet needs. Future studies should include cost-benefit analysis to support the implementation of adjuncts in T1D management.

This unmet-needs analysis may guide clinicians to consider how patient perspectives influence treatment satisfaction. This work may also inform clinical trial design, and calls for greater use of patient-reported outcome measures in clinical trials. Careful consideration of patient perspective may enable more personalized treatment, and may translate to better metabolic outcomes, treatment satisfaction and overall wellbeing in individuals with T1D.

## Supplemental Material

sj-docx-1-jpx-10.1177_23743735241257811 - Supplemental material for Can Unmet Needs Be Addressed by Adjunctive Therapies? Findings from a Patient Perspectives Survey in Adults with Type 1 Diabetes Supplemental material, sj-docx-1-jpx-10.1177_23743735241257811 for Can Unmet Needs Be Addressed by Adjunctive Therapies? Findings from a Patient Perspectives Survey in Adults with Type 1 Diabetes by Bella D. Lamaro, Jerry R. Greenfield and Jennifer R. Snaith in Journal of Patient Experience

sj-docx-2-jpx-10.1177_23743735241257811 - Supplemental material for Can Unmet Needs Be Addressed by Adjunctive Therapies? Findings from a Patient Perspectives Survey in Adults with Type 1 Diabetes Supplemental material, sj-docx-2-jpx-10.1177_23743735241257811 for Can Unmet Needs Be Addressed by Adjunctive Therapies? Findings from a Patient Perspectives Survey in Adults with Type 1 Diabetes by Bella D. Lamaro, Jerry R. Greenfield and Jennifer R. Snaith in Journal of Patient Experience

sj-docx-3-jpx-10.1177_23743735241257811 - Supplemental material for Can Unmet Needs Be Addressed by Adjunctive Therapies? Findings from a Patient Perspectives Survey in Adults with Type 1 Diabetes Supplemental material, sj-docx-3-jpx-10.1177_23743735241257811 for Can Unmet Needs Be Addressed by Adjunctive Therapies? Findings from a Patient Perspectives Survey in Adults with Type 1 Diabetes by Bella D. Lamaro, Jerry R. Greenfield and Jennifer R. Snaith in Journal of Patient Experience

sj-docx-4-jpx-10.1177_23743735241257811 - Supplemental material for Can Unmet Needs Be Addressed by Adjunctive Therapies? Findings from a Patient Perspectives Survey in Adults with Type 1 Diabetes Supplemental material, sj-docx-4-jpx-10.1177_23743735241257811 for Can Unmet Needs Be Addressed by Adjunctive Therapies? Findings from a Patient Perspectives Survey in Adults with Type 1 Diabetes by Bella D. Lamaro, Jerry R. Greenfield and Jennifer R. Snaith in Journal of Patient Experience

sj-docx-5-jpx-10.1177_23743735241257811 - Supplemental material for Can Unmet Needs Be Addressed by Adjunctive Therapies? Findings from a Patient Perspectives Survey in Adults with Type 1 Diabetes Supplemental material, sj-docx-5-jpx-10.1177_23743735241257811 for Can Unmet Needs Be Addressed by Adjunctive Therapies? Findings from a Patient Perspectives Survey in Adults with Type 1 Diabetes by Bella D. Lamaro, Jerry R. Greenfield and Jennifer R. Snaith in Journal of Patient Experience
