# Supplementary figures and images for: Can Unmet Needs Be Addressed by Adjunctive Therapies? Findings from a Patient Perspectives Survey in Adults with Type 1 Diabetes
Source: J Patient Exp. 2024 May 25;11:23743735241257811. doi: 10.1177/23743735241257811 (PMC11128168; doi:10.1177/23743735241257811)

## Supplement 2: Survey Questions


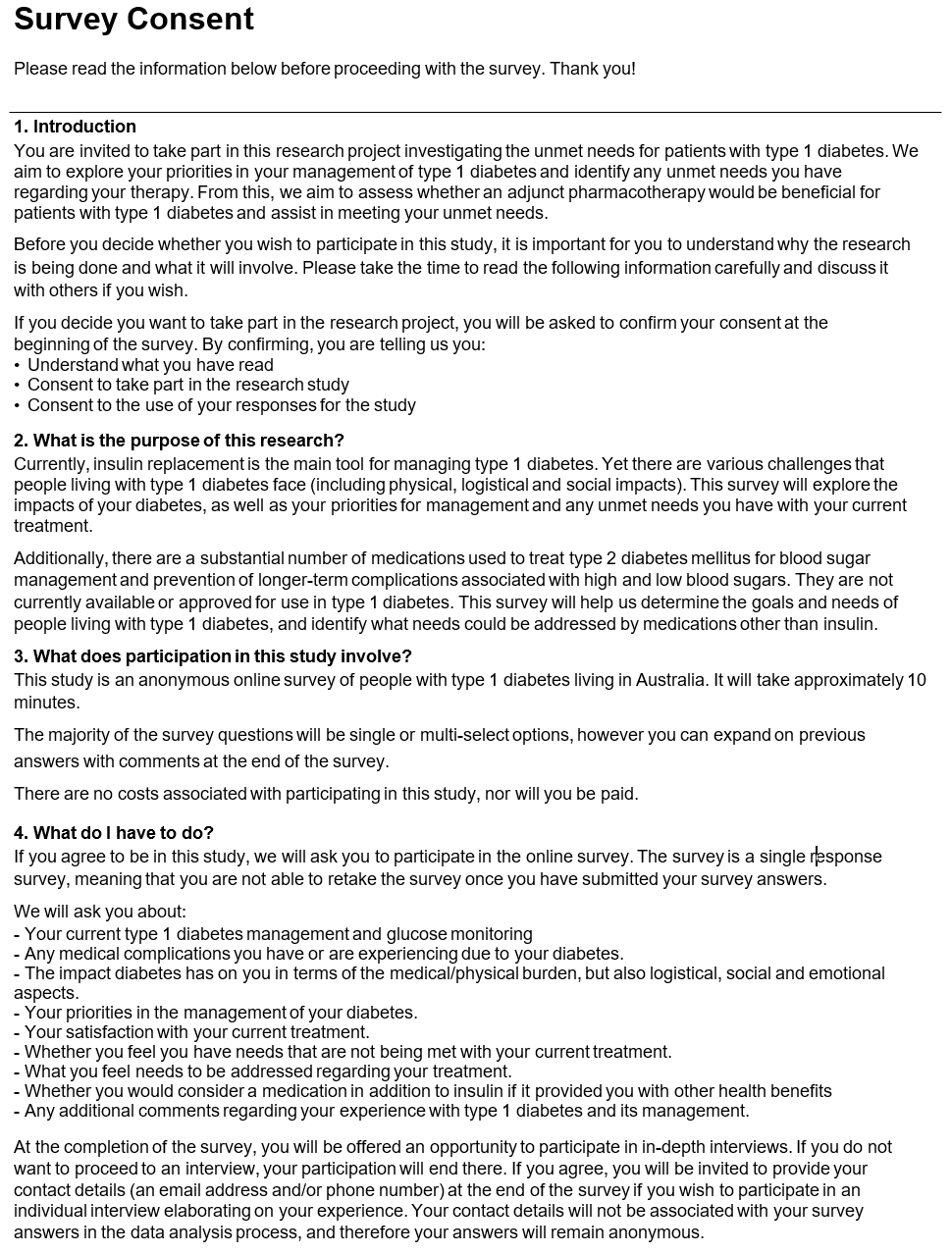


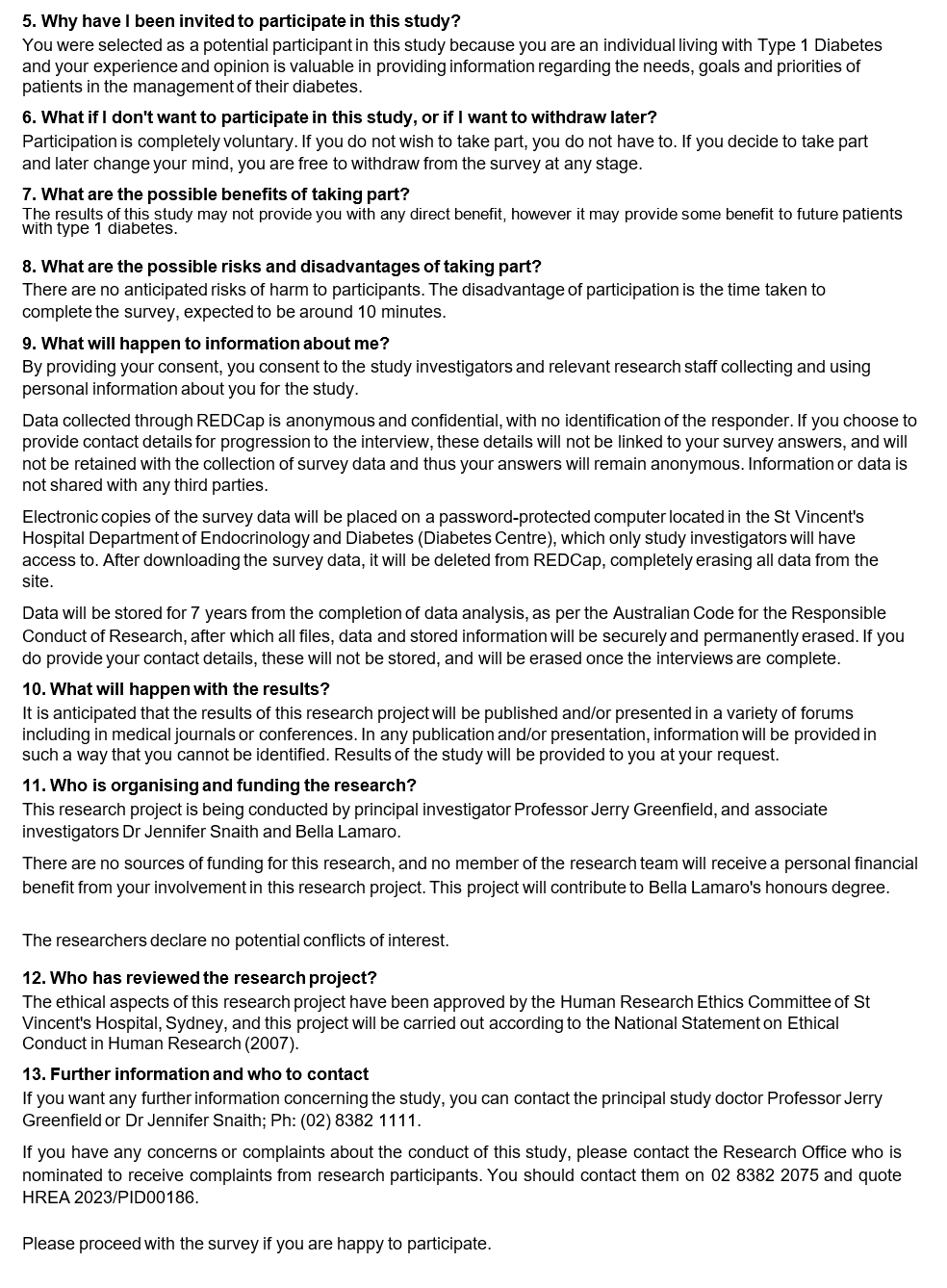


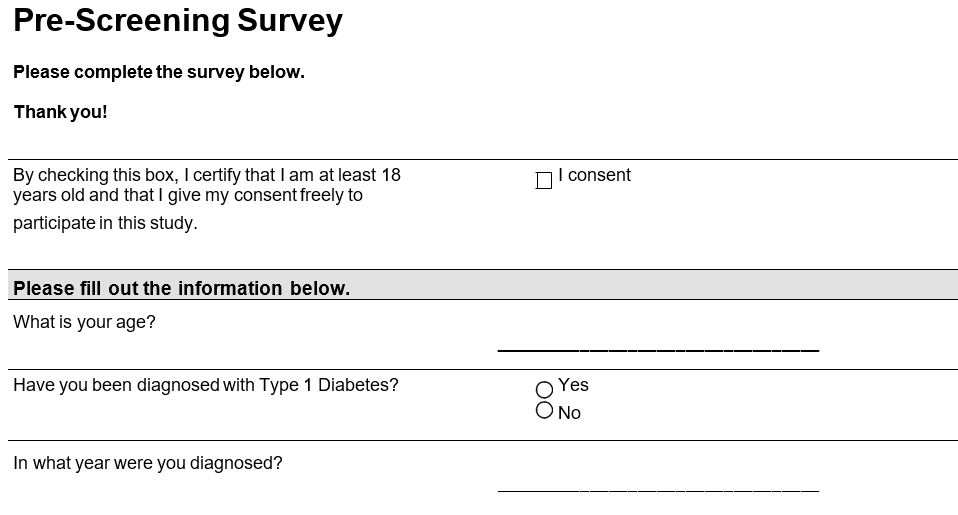


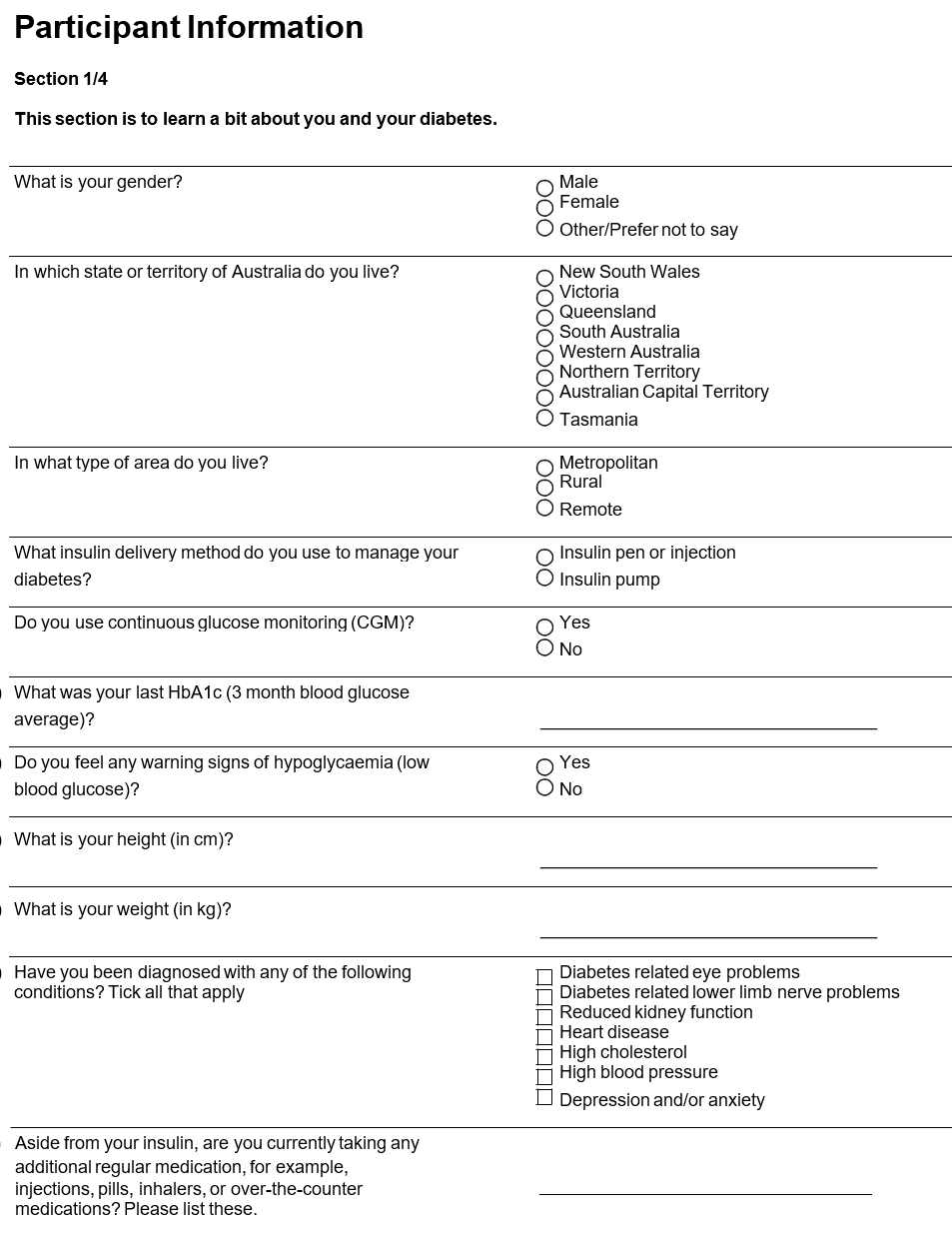


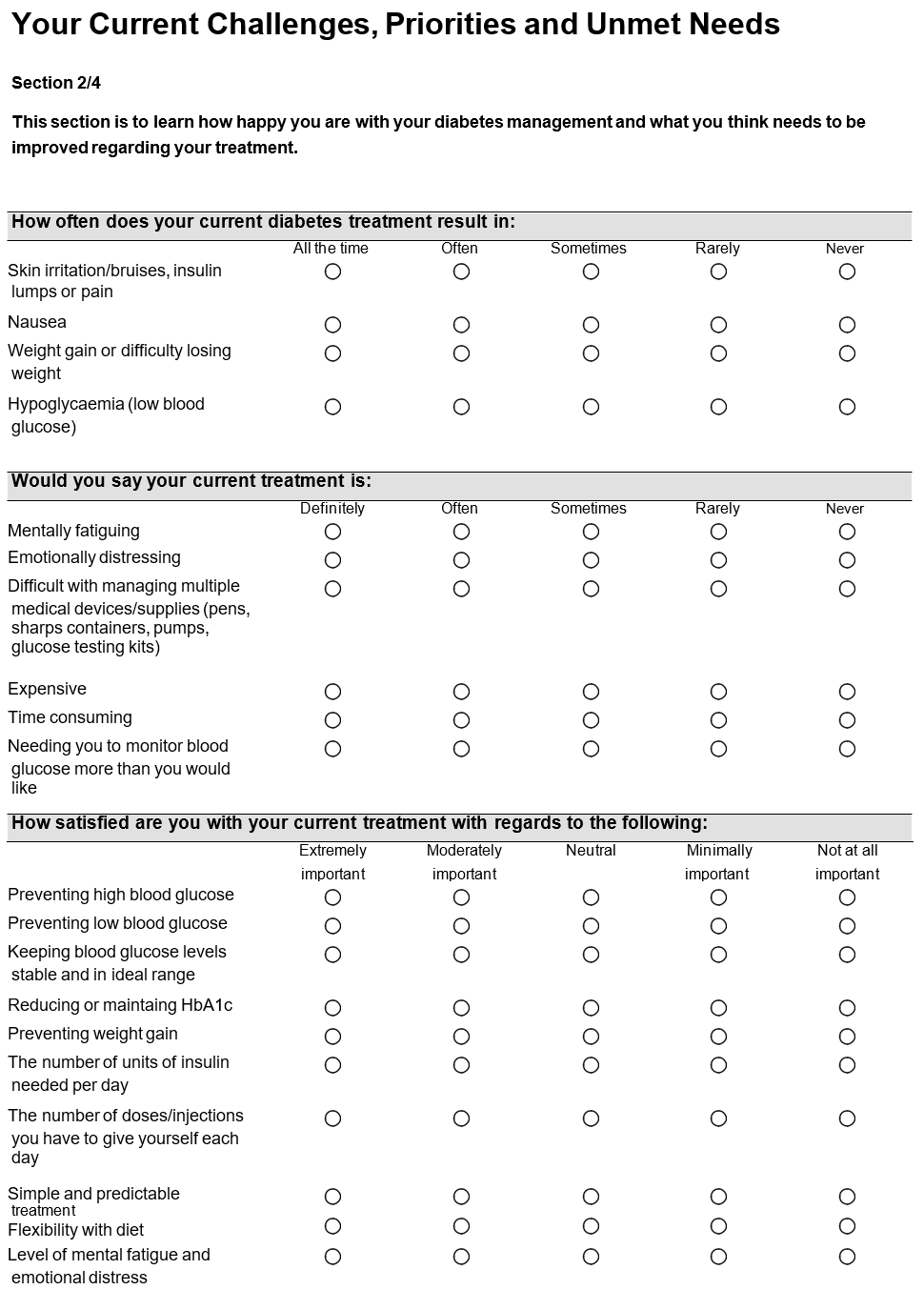


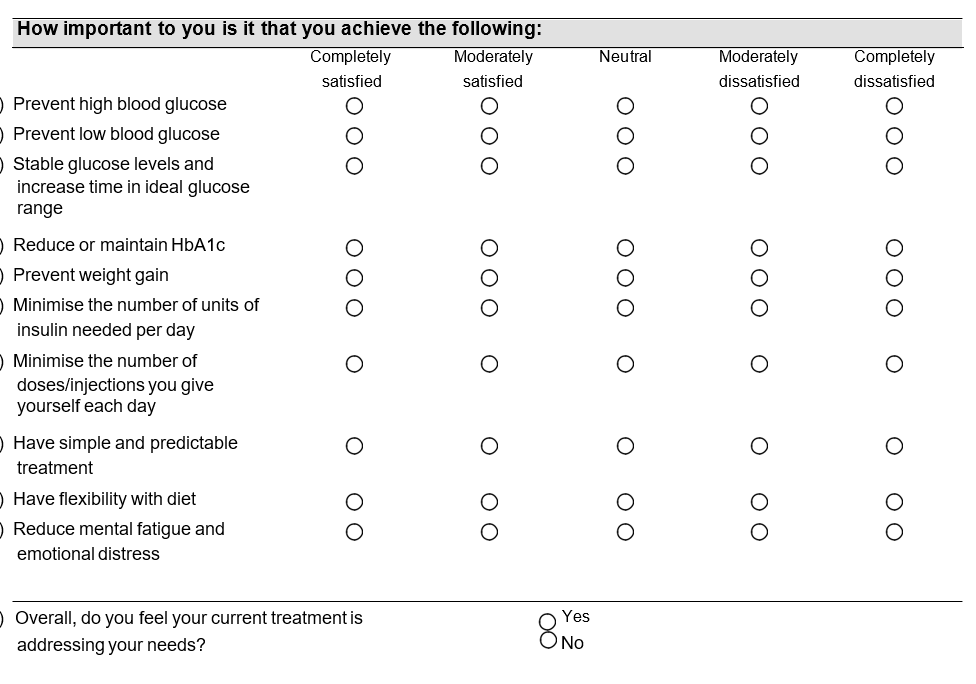


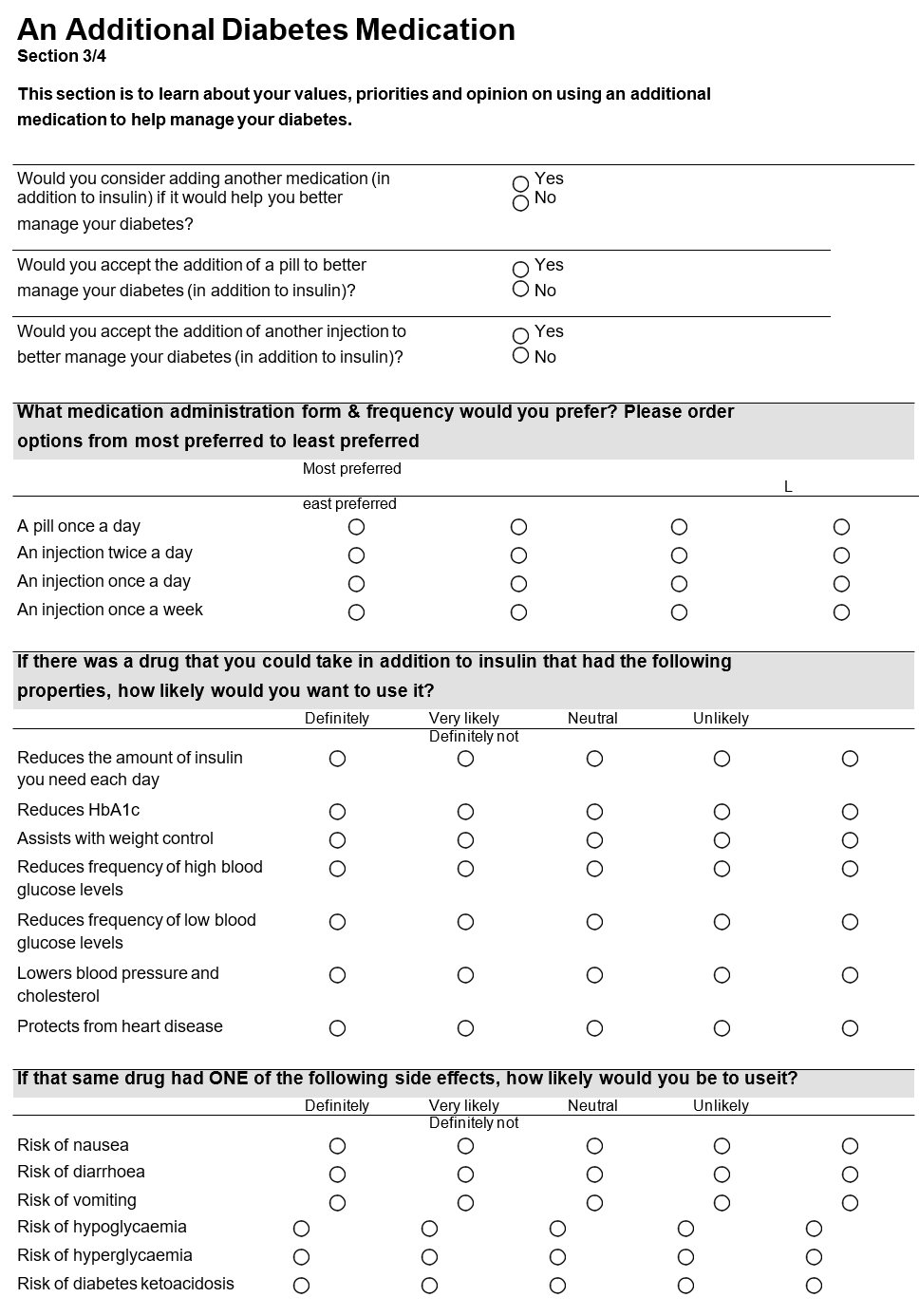


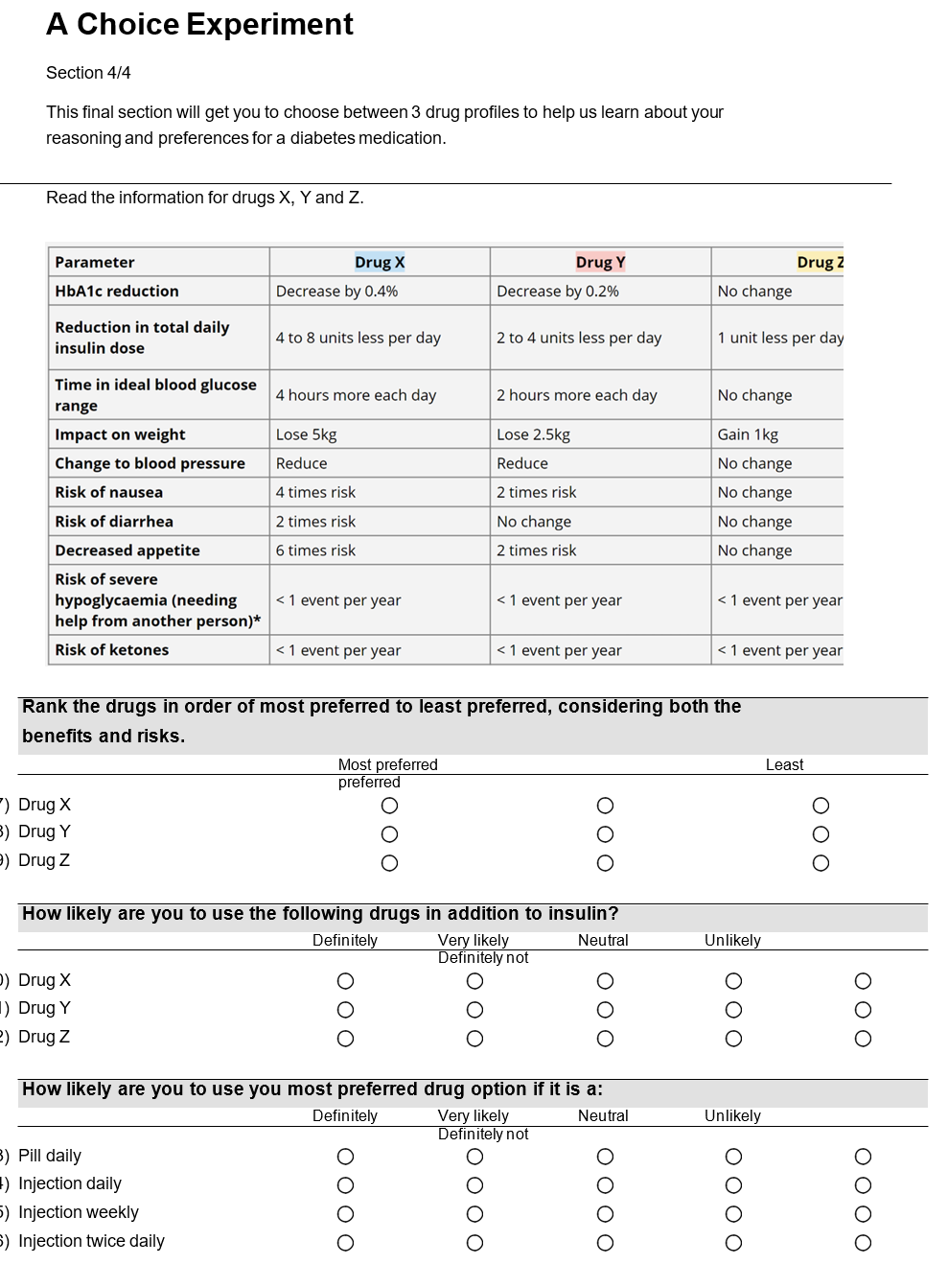


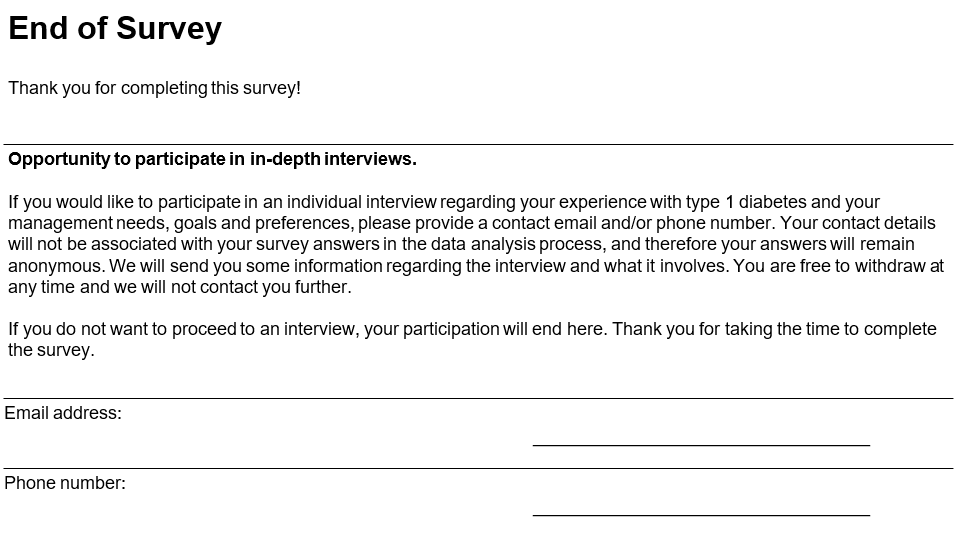

Supplement: sj-docx-2-jpx-10.1177_23743735241257811 - Supplemental material for Can Unmet Needs Be Addressed by Adjunctive Therapies? Findings from a Patient Perspectives Survey in Adults with Type 1 Diabetes [file sj-docx-2-jpx-10.1177_23743735241257811.docx]
