# Supplementary material for: Can Unmet Needs Be Addressed by Adjunctive Therapies? Findings from a Patient Perspectives Survey in Adults with Type 1 Diabetes
Source: J Patient Exp. 2024 May 25;11:23743735241257811. doi: 10.1177/23743735241257811 (PMC11128168; doi:10.1177/23743735241257811)
Supplement: sj-docx-3-jpx-10.1177_23743735241257811 - Supplemental material for Can Unmet Needs Be Addressed by Adjunctive Therapies? Findings from a Patient Perspectives Survey in Adults with Type 1 Diabetes [file sj-docx-3-jpx-10.1177_23743735241257811.docx]

**Supplement 3: Drug profiles used for conjoint risk-benefit analysis, masked to drug, route and dose.** Efficacy and side-effect data derived from phase-three trials of 1.8mg liraglutide (Drug X), 0.6mg liraglutide (Drug Y) and placebo (Drug Z) in T1D^11,12^.

| **Parameter** | **Drug X** | **Drug Y** | **Drug Z** |
| --- | --- | --- | --- |
| **HbA1c reduction** | Decrease by 0.4% | Decrease by 0.2% | No change |
| **Reduction in total daily insulin dose** | 4 to 8 units less per day | 2 to 4 units less per day | 1 unit less per day |
| **Time in ideal blood glucose range** | 4 hours more each day | 2 hours more each day | No change |
| **Impact on weight** | Lose 5kg | Lose 2.5kg | Gain 1kg |
| **Change to blood pressure** | Reduce | Reduce | No change |
| **Risk of nausea** | 4 times risk | 2 times risk | No change |
| **Risk of diarrhea** | 2 times risk | No change | No change |
| **Decreased appetite** | 6 times risk | 2 times risk | No change |
| **Risk of severe hypoglycemia ^a^** | < 1 event per year | < 1 event per year | < 1 event per year |
| **Risk of ketones** | < 1 event per year | < 1 event per year | < 1 event per year |

HbA1c, glycated hemoglobin; kg, kilogram

^a^ defined as requiring external assistance.
