# Supplementary material for: Can Unmet Needs Be Addressed by Adjunctive Therapies? Findings from a Patient Perspectives Survey in Adults with Type 1 Diabetes
Source: J Patient Exp. 2024 May 25;11:23743735241257811. doi: 10.1177/23743735241257811 (PMC11128168; doi:10.1177/23743735241257811)
Supplement: sj-docx-4-jpx-10.1177_23743735241257811 - Supplemental material for Can Unmet Needs Be Addressed by Adjunctive Therapies? Findings from a Patient Perspectives Survey in Adults with Type 1 Diabetes [file sj-docx-4-jpx-10.1177_23743735241257811.docx]

## Supplement 4: Interview Guide

Obtain consent for use of transcript.

Can you please tell me your full name and age?

I’ll start by asking you some questions about your diabetes and management.

- When were you diagnosed with diabetes/ how long have you had diabetes? (Mean duration)
- Do you know your current/last HbA1c level? What was it and when was that taken? (mean)
- What insulin delivery method do you use to manage your diabetes? *(Insulin pen/multiple daily injections vs insulin pump vs insulin pump with automatic adjustments?*
  - How many units of insulin do you take each day, on average?
  - How many adjustments do you have to make?
- I’m going to ask a few questions about any medication you are taking.
  - Any tablets daily? How many?
  - Any daily injections? How many?
  - Are you on any anti-diabetes medication currently?
- Do you use continuous glucose monitoring (CGM)?
- Hypo frequency and awareness.

**We are going to talk a bit about your current treatment and how you feel about it.**

- What are your priorities & goals in the management of your diabetes?
  - *Free answer then subcategories* 1) Day to Day management 2) Long term management
- How satisfied are you with your current treatment?
  - Are you satisfied with your current blood glucose levels on insulin on a day-to-day basis?
  - Are you satisfied with the insulin dosage/number of injections needed each day?
  - Are you satisfied with the level of simplicity/predictability of your diabetes management?
  - Are you satisfied with your weight management with diabetes?
- Have you had any experiences with negative side-effects of insulin use? *(Hypoglycemia, weight gain, injection-site SEs)*
- Do you feel your current treatment addresses your needs?
  - If no: What needs do you feel are not being met with your current treatment?
- What do you feel needs to be addressed/improved regarding your treatment?
  - Do you have any suggestions for this?
  - *If need a prompt: simplify treatment regime, less injections, less painful form of administration, better glucose management, better prevention of hypoglycemia, prevent weight gain.*

**SEGWAY INTO MEDICATION QUESTIONS**

- If there was a medication that you could take in addition to insulin that would help you to better manage your diabetes, would you consider trying it?
  - Why/why not?
  - What ***attributes***/***health benefits*** would it need to have to make you want to use it?
- Considering the following benefits in isolation:
  - If it kept your blood glucose levels within an optimal range, would you consider trying it?
  - If it reduced the amount of insulin you need each day, would you consider trying it?
  - If the medication assisted with weight management, would you use it? Why/why not?
  - If the medication had cardiovascular benefits (improved your heart health, blood pressure and cholesterol), would you use it? Why/why not?
- What side-effects would make you less likely or unwilling to use such a medication (if it had all of the proven benefits we talked about)
  - If it had the best possible outcome to you, would you take the medication at the risk of having those side-effects (*specify what respondent has said*)?
  - What side-effects would you be willing to be at risk of experiencing?
  - *(Do some scenarios)* Nausea, diarrhea, bloated painful after eating, vomiting.
- What do you take into account when you balance the benefits and risks of a medication?
- Does the method of administration have a role in your choice to use a medication i.e., tablet vs injection?
  - Would you prefer an oral tablet than or injection?
  - Does dosage frequency matter to you?
  - Would you prefer a daily tablet, or a weekly injection (why?)
- To summarize, what benefits would you need there for this diabetes medication to be to outweigh any risks or just the effort of taking an additional medication to insulin.

**Relative Importance Scaling: Point Allocation Exercise**

*"During this exercise, we will be allocating points or percentages to both the benefits and risks associated with a diabetes medication that has potential for use in T1D (GLP-1 receptor agonist). Your allocations will help us understand your preferences and priorities when considering these attributes.*

*You have a budget of 100 to make up your ideal medication.*

*When you allocate points to a benefit (green), it indicates that you desire and find it important. So, a higher number = a benefit you want more in a medication. A score of 0 means this benefit isn’t important to you at all.*

*On the other hand, when you allocate points to a risk (red), it signifies that it is important to you to avoid or minimize that risk. So, a higher score for a risk = more deterring for you. A score of 0 means you don’t care about this potential side-effect.*

*The goal is to capture your overall assessment of the benefits and risks and how they influence your decision-making process regarding the medication.”*

Final comments

- Do you have any questions for me or anything you would like to add?
- If you think of anything in the next few days to add please feel free to contact me
