# Supplementary material for: Can Unmet Needs Be Addressed by Adjunctive Therapies? Findings from a Patient Perspectives Survey in Adults with Type 1 Diabetes
Source: J Patient Exp. 2024 May 25;11:23743735241257811. doi: 10.1177/23743735241257811 (PMC11128168; doi:10.1177/23743735241257811)
Supplement: sj-docx-5-jpx-10.1177_23743735241257811 - Supplemental material for Can Unmet Needs Be Addressed by Adjunctive Therapies? Findings from a Patient Perspectives Survey in Adults with Type 1 Diabetes [file sj-docx-5-jpx-10.1177_23743735241257811.docx]

**Supplement 5: Glucagon-like Peptide-1 Receptor Agonist risk and benefit attributes ranked by point allocation.**

| **Attribute** | **Average Point Allocation** |
| --- | --- |
| Optimize BGL stability and increase time-in-range | 27.2 |
| Optimize HbA1c | 16.1 |
| Cardiovascular benefits | 10.5 |
| Reduce insulin requirements | 9.6 |
| Weight loss/management | 9.1 |
| Risk of diabetic ketoacidosis | 7.1 |
| Risk of diarrhea | 6.4 |
| Risk of nausea | 5.4 |
| Risk of hypoglycemia | 5.0 |
| Risk of hyperglycemia | 3.9 |
| **TOTAL POINT BUDGET** | **100** |

BGL, blood glucose level; HbA1c, glycated hemoglobin.
